# Inhibiting repulsive guidance molecule-a suppresses secondary progression in mouse models of multiple sclerosis

**DOI:** 10.1038/s41419-018-1118-4

**Published:** 2018-10-17

**Authors:** Shogo Tanabe, Yuki Fujita, Kaori Ikuma, Toshihide Yamashita

**Affiliations:** 1 Department of Molecular Neuroscience, World Premier International, Immunology Frontier Research Center, Osaka University, 3-1 Yamadaoka, Suita-shi, Osaka 565-0871 Japan; 2 Graduate School of Medicine, Osaka University, 2-2 Yamadaoka, Suita-shi, Osaka 565-0871 Japan; 3 Graduate School of Frontier Biosciences, Osaka University, 1-3 Yamadaoka, Suita-shi, Osaka 565-0871 Japan

## Abstract

Multiple sclerosis (MS) is an autoimmune disease of the central nervous system that is characterized by motor deficits, fatigue, pain, cognitive impairment, and sensory and visual dysfunction. Secondary progressive multiple sclerosis (SPMS) is a progressive form of MS that develops from relapsing-remitting MS. Repulsive guidance molecule-a (RGMa) has diverse functions, including axon growth inhibition and immune regulation. Here, we show inhibiting RGMa had therapeutic effects in mouse models of SPMS. We induced experimental autoimmune encephalomyelitis in nonobese diabetic mice (NOD-EAE mice) and treated them with humanized anti-RGMa monoclonal antibody. This treatment significantly suppressed secondary progression of disease and inflammation, demyelination and axonal degeneration. In addition, treatment with anti-RGMa antibody promoted the growth of corticospinal tracts and motor recovery in targeted EAE mice with inflammatory lesions in the spinal cord. Collectively, these results show that a humanized anti-RGMa antibody has therapeutic effects in mouse models of SPMS.

## Introduction

Multiple sclerosis (MS) is an autoimmune disease characterized by inflammation, demyelination, and axonal degeneration in the central nervous system (CNS)^1,2^. The clinical course of MS is highly heterogeneous, but can be broadly classified into relapsing and progressive forms. Approximately 85% of MS patients exhibit relapsing-remitting MS (RRMS), a biphasic course with alternating episodes of neurological disability and recovery. Within 10 years, 50% of RRMS patients develop secondary progressive MS (SPMS), which is characterized by progressive neurological decline^3^.

Currently, there are only a few therapeutic options available to patients with MS. Ocrelizumab has therapeutic effects for primary progression of MS, which is characterized by progressive neurological deficit without relapsing-remitting^4,5^. Siponimod has recently been shown to reduce the progression of disabilities in patients with SPMS in clinical trials^4,6^. Mitoxantron is approved for SPMS treatment with significant toxicity^7^. However, these treatments are not able to halt disease progression of SPMS without toxicity, or recover the neurological deficits. While immunosuppressive treatments have therapeutic effects on RRMS, many of them are not effective in patients with SPMS^8^. Therefore, immune regulation is not sufficient for suppression of secondary progression. MRI imaging and histological analysis revealed that neurodegeneration is a pathological hallmark of SPMS^9–11^. In addition, progressed neurodegeneration resulted in the atrophy of brain and spinal cord of SPMS patients^12^, suggesting that neurodegeneration is a key to treat disease progression of SPMS.

Repulsive guidance molecule-a (RGMa) is a glycosylphosphatidylinositol-anchored membrane protein, and plays a crucial role in the neural network formation by binding with neogenin, which is a receptor for RGMa^13^. It has been reported that expression of RGMa is upregulated in CNS of MS patients^14^. We previously showed RGMa contributes to pathogenesis of experimental autoimmune encephalomyelitis (EAE), an animal model of MS. RGMa exacerbates inflammation by activating T cells in the CNS of EAE mice^15^. RGMa expressed in T cells induces neurodegeneration^16^. RGMa inhibition with anti-RGMa antibody treatment attenuates clinical manifestations in EAE mice. Notably, anti-RGMa antibody treatment promotes neuroregeneration and neuroprotection in targeted EAE, a focal model of EAE in rats^14^. These evidences indicate inhibition of RGMa has clinical effects for EAE by diverse mechanisms such as suppressing inflammation, neurodegeneration, and promoting neuroregeneration. However, it remains unknown whether inhibition of RGMa can provide therapeutic benefits in SPMS.

Based on these prior findings, we hypothesized that RGMa inhibition has therapeutic effects for secondary progression of EAE. In the present study, we examined whether a humanized monoclonal antibody against RGMa^17^ would have therapeutic effects on secondary progression of EAE in nonobese diabetic mice, an experimental model that closely resembles SPMS. We also developed targeted EAE mice to observe the effects of the humanized anti-RGMa antibody on neuroregeneration and functional recovery.

## Results

### Humanized anti-RGMa antibody specifically recognizes RGMa proteins

**Fig. 1:**
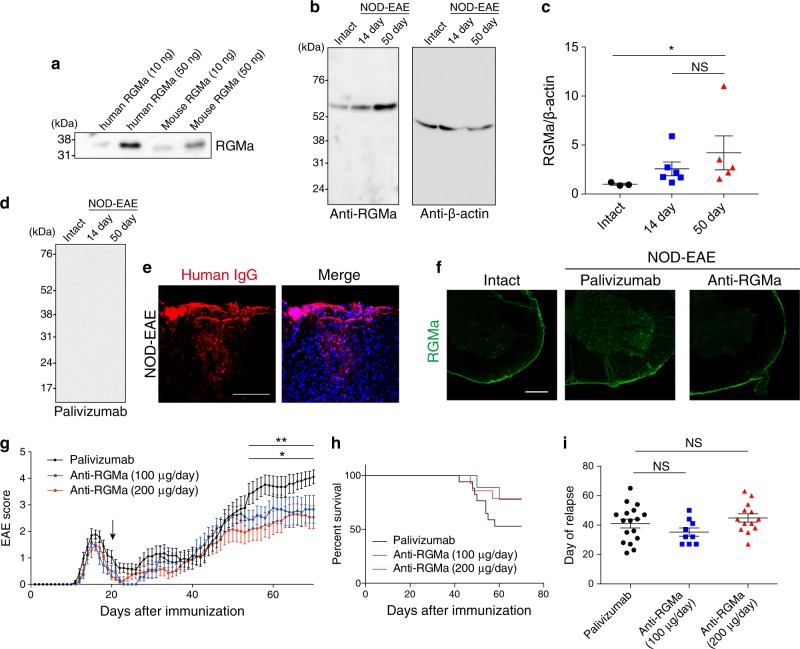
Treatment with humanized anti-RGMa antibody prevents secondary disease progression in NOD-EAE mice. **a** 10 or 50 ng of mouse and human recombinant RGMa proteins were subjected to SDS-PAGE and western blotting with humanized anti-RGMa antibody. **b** Spinal cord lysates from intact, NOD-EAE mice at 14 days post-immunization and NOD-EAE mice at 50 days post-immunization were subjected to SDS-PAGE and western blotting with humanized anti-RGMa or β-actin antibody. Left numbers show the molecular weight (kDa). **c** Quantification analysis of RGMa expression in the spinal cord of intact (*n* = 3), NOD-EAE mice at 14 days post-immunization (*n* = 6) and NOD-EAE mice at 50 days post-immunization (*n* = 5). Band intensity of RGMa was normalized to that of β-actin. Statistical analysis was performed by Kruskal−Wallis ANOVA test followed by Dunn’s test. **d** Spinal cord lysates from intact, NOD-EAE mice at 14 days post-immunization, and 50 days post-immunization were subjected to western blotting with palivizumab. **e** Representative images show the infiltrating human IgG stained with specific anti-human IgG (red) in the spinal cord of NOD-EAE mice at 70 days after immunization. Counterstaining was performed with DAPI (blue). Scale bar, 50 μm. **f** Spinal cord sections from control or NOD-EAE mice at 70 days after immunization treated with palivizumab or humanized anti-RGMa antibody were stained with rabbit anti-RGMa antibody. Scale bar: 250 μm. Images are representative of spinal cords extracted from at least three mice per treatment group. **g** Palivizumab or anti-RGMa antibodies were intravenously injected every 3 days after 20 days of immunization into NOD-EAE mice. EAE clinical scores were determined for NOD-EAE mice treated with palivizumab (200 μg/day, *n* = 17, black), anti-RGMa (100 μg/day, *n* = 9, blue) and anti-RGMa (200 μg/day, *n* = 14, red). The arrow indicates the time (20 days after immunization) when the antibody treatment was started. Statistical analysis was performed by two-way ANOVA followed by Bonferroni tests. *palivizumab vs. anti-RGMa (100 μg/day), **palivizumab vs. anti-RGMa (200 μg/day). (**h**) Survival curves of NOD-EAE mice treated with palivizumab or anti-RGMa antibody. (**i**) The days after immunization at which relapse occurred for NOD-EAE mice treated with palivizumab or anti-RGMa. Statistical analysis was performed by one-way ANOVA followed by the Tukey−Kramer test. NS not significant. *p *<* 0.05, **p *<* 0.01

We first induced chronic progressive EAE by immunizing nonobese diabetic (NOD) mice with myelin oligodendrocyte glycoprotein (MOG) peptide (NOD-EAE)^18^. NOD-EAE mice exhibited the first acute symptoms 12–16 days after immunization with subsequent remission within 20 days. Secondary progression was then observed approximately 35−45 days after immunization. In this study, we used a humanized, monoclonal, anti-human RGMa antibody^17^, which recognizes both human and mouse recombinant RGMa proteins (Fig. 1a). The antibody recognized RGMa specifically in the spinal cord of both control and NOD-EAE mice. The expression of RGMa appears to be increased in the spinal cord of NOD-EAE mice at the secondary progressive phase (50 days post-immunization) when compared with control mice, but did not change compared with the acute phase (14 days post-immunization) (Fig. 1b, c), suggesting involvement of RGMa in the secondary disease progression in NOD-EAE mice.

To validate the effect of anti-RGMa antibody for inhibiting RGMa in CNS of NOD-EAE, we intravenously injected the antibody into NOD-EAE mice from 20 days after immunization. Palivizumab is a humanized monoclonal antibody against the protein of respiratory syncytial virus, and is IgG1κ isotype, which is the same isotype as anti-RGMa antibody. Moreover, palivizumab has no therapeutic effects for EAE, and did not react with nonspecific proteins in the spinal cord of control and NOD-EAE mice (Fig. 1d). Thus, we used palivizumab as a control. We observed infiltration of administered human antibody in inflammatory lesion (Fig. 1e) and decreased expression of RGMa in the spinal cord of NOD-EAE mice after treatment with the humanized RGMa antibody (Fig. 1f). This result suggests that the humanized anti-RGMa antibody efficiently depleted RGMa or led to decreased expression of RGMa in the spinal cord.

### Anti-RGMa antibody prevents secondary progression of NOD-EAE

To examine whether the anti-RGMa antibody prevents secondary disease progression in NOD-EAE mice, we observed EAE score daily and found that anti-RGMa antibody treatments inhibited increasing EAE score after 50 days of immunization, suggesting that anti-RGMa prevented secondary progression in NOD-EAE mice (Fig. 1g, Table 1). Anti-RGMa antibody treatment also appeared to prolong the survival of NOD-EAE mice compared to palivizumab-treated NOD-EAE mice, although this effect was not statistically significant because of low mortality rate of NOD-EAE mice (Fig. 1h). In contrast, this treatment did not affect the day of relapse (Fig. 1i), suggesting that RGMa does not regulate the onset of secondary progression. These results indicate that anti-RGMa antibody prevented secondary progression in NOD-EAE mice.

**Table 1:** EAE scores of mice treated with control or anti-RGMa antibodies

Treatment group	Mean relapse days	Mean disease score between 20 and 49 days	Mean disease score after 50 days	Score above 3.0 at 70 days
Palivizumab	41.1 ± 3.0	1.15 ± 0.23	3.57 ± 0.35	14/17 (82.3%)
Anti-RGMa (100 μg)	35.2 ± 2.8	0.94 ± 0.19	2.69 ± 0.42	4/9 (44.4%)
Anti-RGMa (200 μg)	44.8 ± 2.8	0.79 ± 0.19	2.32 ± 0.41	5/14 (35.7%)

Mean ± SEM

### RGMa is involved in inflammation, axonal loss, and demyelination in secondary progression of NOD-EAE mice

**Fig. 2:**
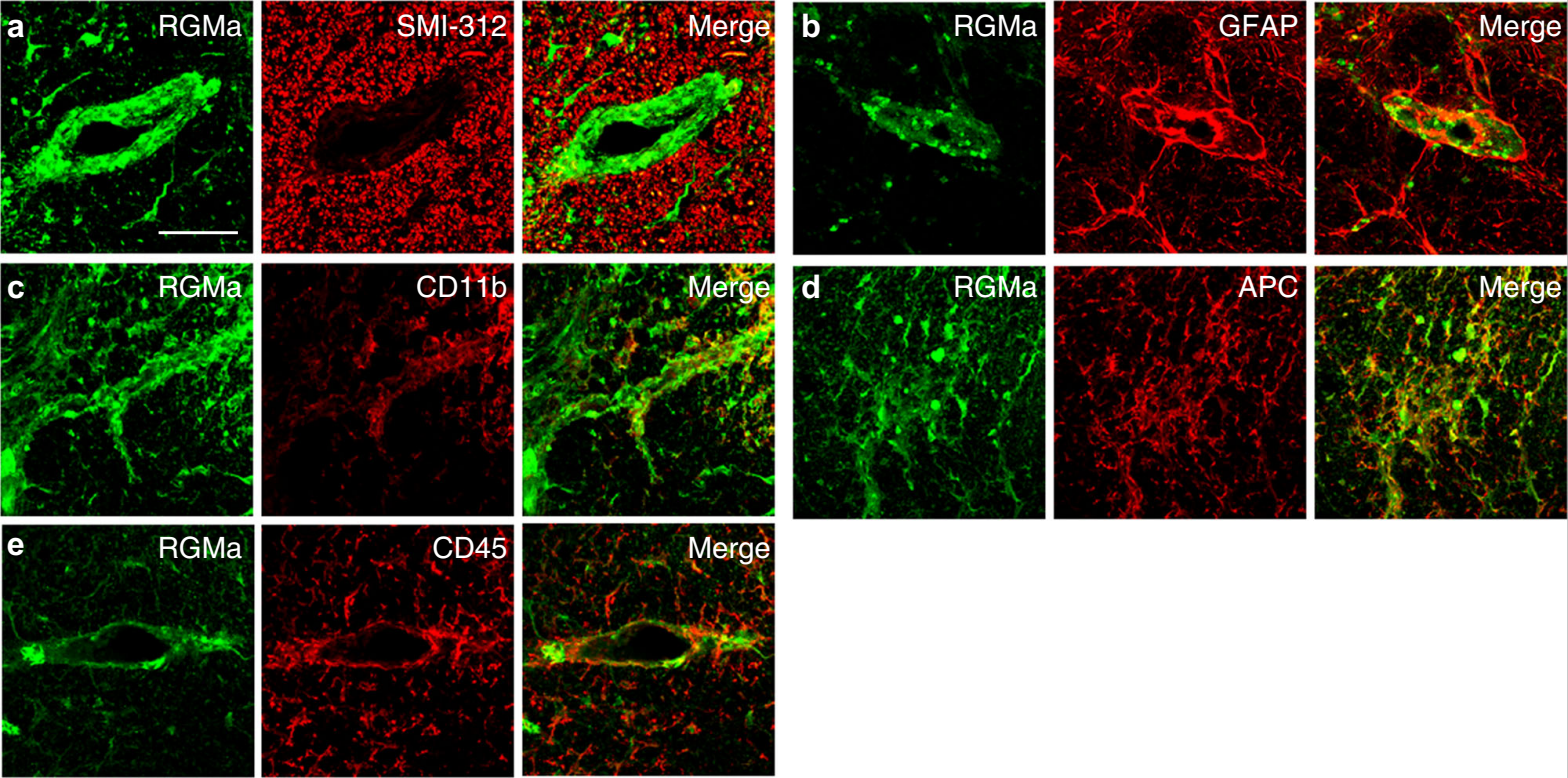
RGMa is expressed in leukocytes and oligodendrocytes of NOD-EAE mice during secondary progression. Dual immunostaining for RGMa with SMI-312 (**a**), GFAP (**b**), CD11b (**c**), APC (**d**), and CD45 (**e**) in the spinal cord of NOD-EAE mice at 70 days after immunization. Scale bar, 50 μm

**Fig. 3:**
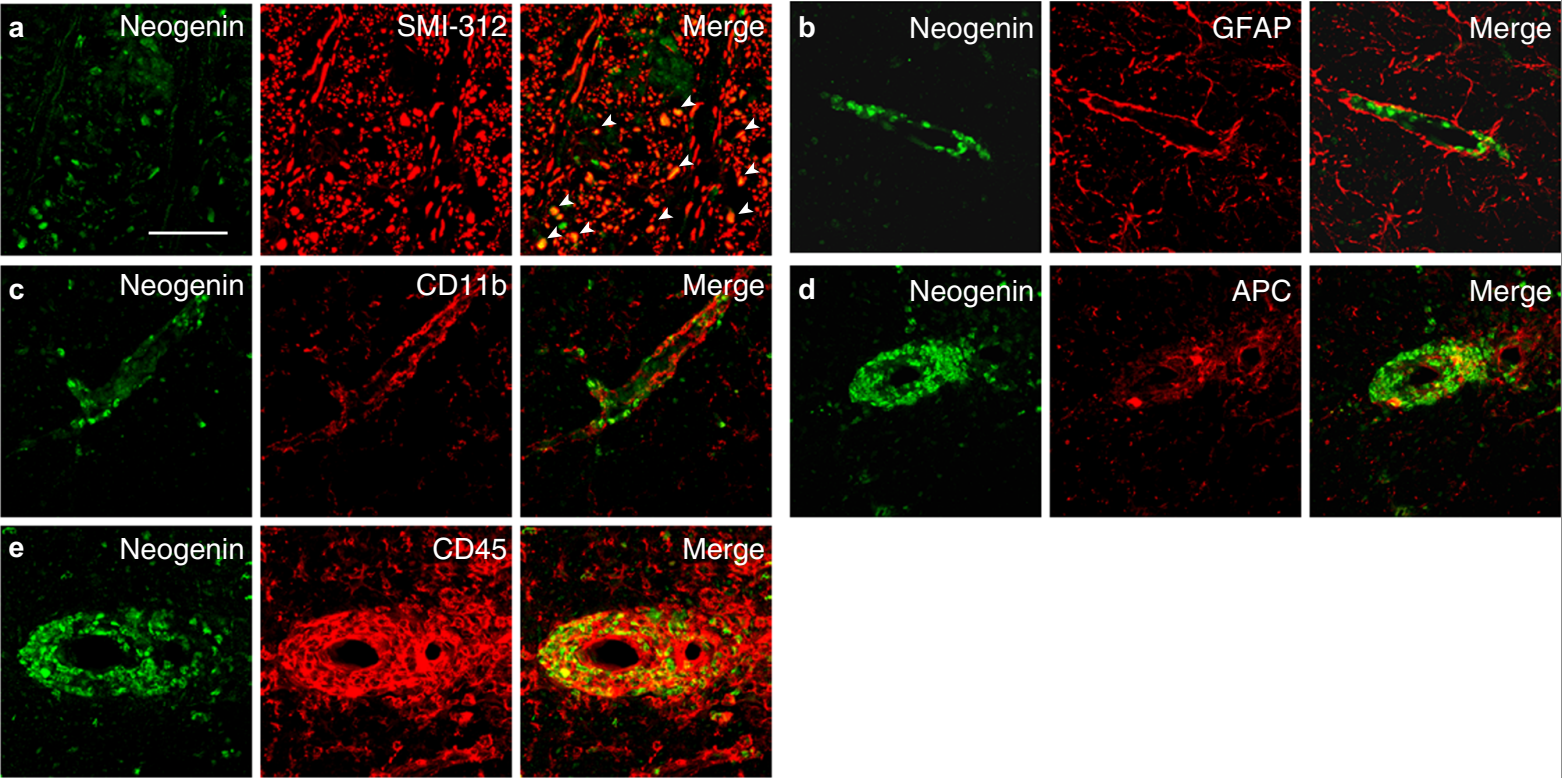
Neogenin is expressed in axons and leukocytes of NOD-EAE mice during secondary progression. **a−e** Dual immunostaining for neogenin with SMI-312 (**a**), GFAP (**b**), CD11b (**c**), APC (**d**), and CD45 (**e**) in the spinal cord of NOD-EAE mice at 70 days after immunization. Scale bar, 50 μm

**Fig. 4:**
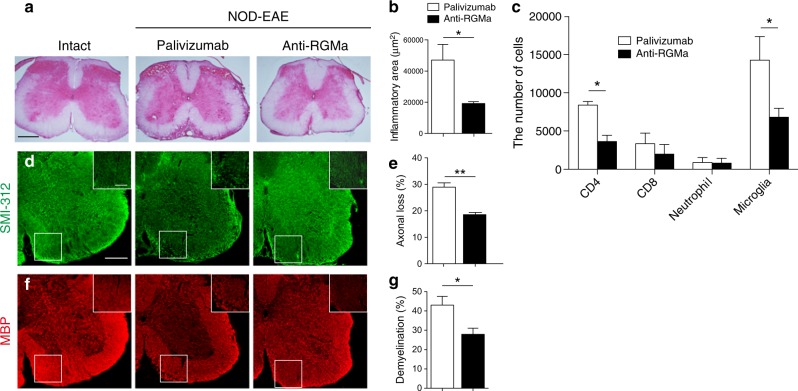
Treatment with humanized anti-RGMa antibody suppresses inflammation, demyelination, and neurodegeneration in NOD-EAE mice. **a** Lumbar spinal cord sections from control mice and NOD-EAE mice treated with palivizumab (EAE score: 4) or anti-RGMa antibody (EAE score: 2) were evaluated by HE staining at 70 days of immunization. Scale bar: 200 μm. Images are representative of spinal cords extracted from at least three mice per treatment group. **b** Quantitation of inflammation as assessed in (**a**) (palivizumab: *n* = 4, anti-RGMa: *n* = 4, assessed by Student’s *t* test). **c** The number of CD4^+^ T cells, CD8^+^ T cells, Ly-6G^+^ neutrophils, and CD45^mid^ CD11b^+^ microglia in the spinal cord of NOD-EAE mice treated with palivizumab or anti-RGMa antibody analyzed by flow cytometry at 70 days of immunization (palivizumab: *n* = 3, anti-RGMa: *n* = 3, assessed by Student’s *t* test). **d** Immunohistochemistry for axonal neurofilaments (SMI-312) from lumbar spinal cord sections of control mice and NOD-EAE mice treated with palivizumab (EAE score: 4) or anti-RGMa antibody (EAE score: 2) at 70 days of immunization. Inset images are a high magnification of the area within the white squares. Scale bar: main image 250 μm; inset 100 μm. **e** Quantitation of axonal loss as assessed in (**d**) (palivizumab: *n* = 4, anti-RGMa: *n* = 4, assessed by Student’s *t* test). **f** Immunohistochemistry for MBP from lumbar spinal cord sections of control and NOD-EAE mice treated with palivizumab (EAE score: 4) or anti-RGMa antibody (EAE score: 2) at 70 days of immunization. **g** Quantification of demyelination as assessed in (**f**) (palivizumab: *n* = 5, anti-RGMa: *n* = 6, assessed by Student’s *t* test). Error bars represent mean ± SEM. *p < 0.05, **p < 0.01

Immunohistochemistry showed RGMa was expressed in CD11b^+^ microglia, APC^+^ oligodendrocytes, and CD45^+^ leukocytes in spinal cord of NOD-EAE mice (Fig. 2a−e), as observed in spinal cord injury or acute phase of EAE^15,19,20^. On the other hand, neogenin, receptor for RGMa, was expressed in SMI-312^+^ axons in inflammatory lesions, and CD45^+^ leukocytes (Fig. 3a−e), as previously shown^16^. These observations prompted us to investigate whether anti-RGMa antibody affected inflammation, axonal degeneration, and demyelination in NOD-EAE mice. To test the effects of anti-RGMa antibody for inflammation, we performed hematoxylin−eosin (HE) staining on spinal cord sections and flow cytometry analysis from NOD-EAE mice at 70 days after immunization. Histolopathological analysis of lumbar spinal cord sections revealed that cell infiltration in NOD-EAE mice was significantly suppressed by anti-RGMa antibody treatment compared to palivizumab-treated NOD-EAE mice (Fig. 4a, b). The number of CD4^+^ T cells and microglia in the spinal cord were significantly reduced by anti-RGMa antibody treatment, as determined by flow cytometric analysis (Fig. 4c). No differences were observed in the number of CD8^+^ cells or neutrophils (Fig. 4c). These results suggest that anti-RGMa antibody suppressed inflammation by inhibiting CD4^+^ T-cell infiltration and microglial activation in NOD-EAE mice.

We also examined whether anti-RGMa antibody inhibited demyelination and neurodegeneration in NOD-EAE mice. Importantly, staining with anti-SMI-312 antibody showed that axonal degeneration in the spinal cord was inhibited by anti-RGMa antibody treatment in NOD-EAE mice (Fig. 4d, e). Staining of spinal cord sections with anti-myelin basic protein (MBP) antibody revealed that anti-RGMa antibody also significantly reduced demyelination (Fig. 4f, g). These results suggest that RGMa also promotes axonal degeneration and demyelination in NOD-EAE mice with the anti-RGMa antibody.

### Anti-RGMa antibody promotes neuroregeneration in targeted EAE mice

**Fig. 5:**
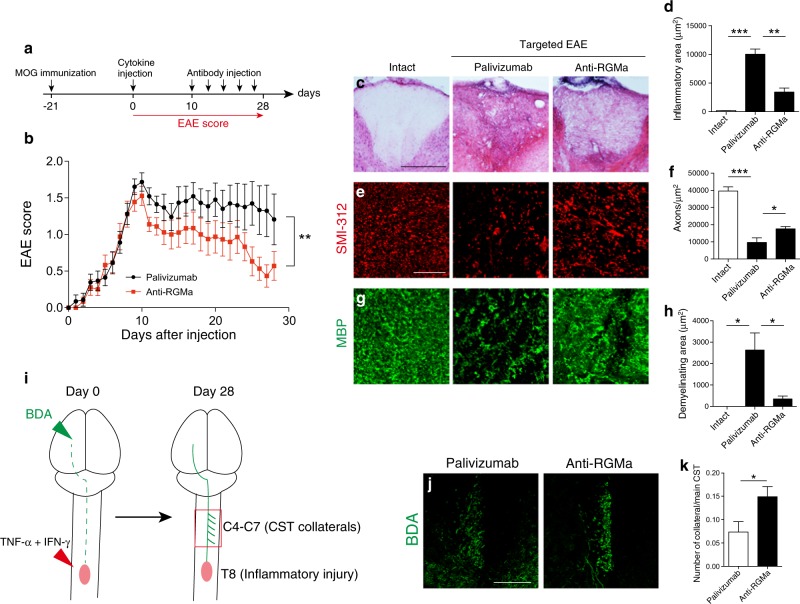
Treatment with humanized anti-RGMa antibody promotes functional recovery and axonal regeneration in a targeted EAE model. **a** A schematic representation of the protocol followed for monitoring targeted EAE progression. **b** The graph shows the time course of the EAE clinical score of targeted EAE mice treated with palivizumab or humanized anti-RGMa antibody (palivizumab; *n* = 23, anti-RGMa; *n* = 18, assessed by the Mann−Whitney test). **c** T8 spinal cord sections from control and targeted EAE mice treated with palivizumab (EAE score: 1.5) or anti-RGMa antibody (EAE score: 0.5) were evaluated by H&E staining at 28 days of immunization. Scale bar: 200 μm. **d** Quantification of inflammation as assessed in (**c**) (control; *n* = 3, palivizumab; *n* *=* 5, anti-RGMa; *n* = 5, assessed by one-way ANOVA followed by the Tukey−Kramer test). **e** Immunohistochemical staining for SMI-312 in T8 spinal cord sections from control and targeted EAE mice treated with palivizumab (EAE score: 1.5) or anti-RGMa antibody (EAE score: 0.5) at 28 days of immunization. Scale bar: 50 μm. **f** Quantification of the number of axons as assessed in (**e**) (control; *n* = 3, palivizumab; *n* *=* 6, anti-RGMa; *n* = 6, assessed by one-way ANOVA followed by the Tukey−Kramer test). **g** Immunohistochemistry for MBP in the dorsal column of T8 spinal cord sections from control and targeted EAE mice treated with palivizumab (EAE score: 1.5) or humanized anti-RGMa antibody (EAE score: 0.5). **h** Quantification of the demyelinating area as assessed in (**g**) (control; *n* = 3, palivizumab; *n* *=* 5, anti-RGMa; *n* = 5, assessed by one-way ANOVA followed by the Tukey−Kramer test). **i** A schematic representation of the axonal remodeling protocol. **j** BDA-labeled hind limb CST axons in C7 spinal cord sections from targeted EAE mice treated with palivizumab (EAE score: 1.5) or anti-RGMa (EAE score: 0.5). Scale bar: 100 μm. **k** Quantification of the number of collaterals as assessed in (**j**) (palivizumab; *n* *=* 6, anti-RGMa; *n* = 7, assessed by Student’s *t* test). Error bars represent mean ± SEM. *p < 0.05, **p < 0.01, ***p < 0.001

We next investigated the effects of our anti-RGMa antibody on neuroregeneration in EAE. As impaired neural network is responsible for the neurological symptoms under SPMS, neural network repair should be one of the effective strategies for the treatment of SPMS. We assessed whether the anti-RGMa antibody could regenerate neural network by promoting axonal sprouting, leading to functional recovery in EAE. We generated a targeted EAE mouse model, in which a single focal inflammatory lesion was generated in the dorsal column of the spinal cord to injure the corticospinal tract (CST), resulting in the impairment of voluntary movement^21^. This model allows us to assess the functional recovery from motor deficit that is correlated to axonal sprouting of the CST. We administered palivizumab or anti-RGMa antibody intravenously into targeted EAE mice at the acute phase of motor deficits and observed EAE scores daily (Fig. 5a). Although targeted EAE mice treated with palivizumab spontaneously recover from motor deficits slowly, anti-RGMa antibody treatment significantly promoted motor recovery (Fig. 5b). This result suggests that anti-RGMa treatment is able to promote functional recovery from motor deficit.

We next examined the effects of anti-RGMa antibody on suppressing inflammation, axonal degeneration, and demyelination in the inflammatory lesion by histological analysis. HE staining showed that anti-RGMa antibody reduced the area of the inflammatory lesion (Fig. 5c, d). Moreover, treatment with the anti-RGMa antibody increased the number of SMI-312^+^ axons (Fig. 5e, f) and reduced demyelination (Fig. 5g, h). In addition to confirming these observations, we found that anti-RGMa antibody suppressed inflammation and axonal loss in targeted EAE mice.

Studies using animal models of EAE have also demonstrated axonal remodeling in response to spinal injury to compensate for lost functions^21,22^. To examine whether anti-RGMa antibody promotes axonal sprouting in our targeted EAE mice, we injected biotin dextran amine (BDA), an anterograde tracer, into the hind limb area of the motor cortex (Fig. 5i). We counted the number of CST collaterals in the cervical spinal cord at day 28 and observed increased axonal branches from the CST upon anti-RGMa antibody treatment (Fig. 5j, k). These results suggest that anti-RGMa antibodies have the capacity to regenerate neural network by promoting axonal sprouting.

## Discussion

In this study, we have demonstrated the therapeutic effects of a humanized anti-RGMa antibody^17^ on secondary progression in NOD-EAE mice. The antibody suppressed inflammation, axonal degeneration, and demyelination. Moreover, the antibody promoted neuroregeneration by repairing the neural network. However, it had no effect on the onset of secondary progression and only partially suppressed progression in our model, suggesting complexities in the mechanisms of SPMS that are not fully dependent on RGMa.

As RGMa has a number of physiological functions and is expressed in various types of cells, the effects of anti-RGMa antibody may be dependent on multiple therapeutic mechanisms. The suppression of inflammation by an anti-RGMa antibody in NOD-EAE mice may be mediated by inhibiting T-cell activation, which is consistent with previous report that demonstrates RGMa enhances T-cell activation and inflammation in EAE^15^. It was recently reported that Eomes^+^ CD4^+^ T cells play essential roles in chronic neuroinflammation in patients with SPMS and chronic EAE mice^23^. In the present study, we showed that an anti-RGMa antibody reduced the number of CD4^+^ T cells in the spinal cord of NOD-EAE mice. Thus, the anti-RGMa antibody may prevent secondary progression by inhibiting the activation of CD4^+^ T cells, including Eomes^+^ CD4^+^ T cells.

Axonal degeneration is a major pathological hallmark of SPMS^11,24^. Accumulating evidence suggests that inhibiting axonal degeneration is key to preventing MS progression^18,25,26^. We have previously shown that neogenin, the RGMa receptor, is expressed on SMI-312^+^ axons, and RGMa-neogenin signaling drives axonal degeneration in EAE^16^. Neogenin was also expressed on SMI-312^+^ axons in NOD-EAE mice in the present study. Moreover, we showed upregulation of RGMa in the spinal cord of NOD-EAE mice during secondary progressive phase. We therefore speculate that RGMa-neogenin signaling mediates axonal degeneration in the progressive phase of EAE. In addition, RGMa regulates T-cell activation^15^. RGMa may induce axonal degeneration at least through two distinct mechanisms that activate T cells and induce axonal degeneration. Indeed, our anti-RGMa antibody effectively suppressed axonal degeneration in NOD-EAE and targeted EAE mice, which is consistent with prior reports that RGMa enhances axonal degeneration in EAE^14,16^.

We also demonstrated that inhibition of RGMa promoted corticospinal growth in targeted EAE mice. RGMa inhibits axonal growth in CNS, and anti-RGMa treatment promotes neuroregeneration in the spinal cord by inhibiting RGMa expressed in oligodendrocytes and microglia^19,20^. In targeted EAE mice, T-cell infiltration was not observed in cervical spinal cord, because we induced a focal inflammatory lesion in the thoracic spinal cord. Therefore, it is suggested that anti-RGMa antibody promotes neuroregeneration by regulating oligodendrocytes and microglia in targeted EAE mice as well. In spinal cord injury and targeted EAE mice, the CST forms collaterals and develops compensatory neural projections in rodents and primates^21,22,27,28^. This spontaneous neural rewiring has been suggested to play a pivotal role in functional recovery after spinal cord injury^28^. It is difficult to determine whether anti-RGMa promotes neural rewiring also in NOD-EAE mice because of diffuse inflammatory lesions. We showed anti-RGMa treatment facilitated repair of the neural network when CST was injured in targeted EAE mice. It is expected that anti-RGMa treatment also contributes to neural rewiring and neuroregeneration in NOD-EAE and SPMS. As an impaired neural network is responsible for the neurological progression of MS^29^, effective neural repair will be essential to prevent the progression of MS.

Collectively, humanized anti-RGMa antibody prevented the progression of motor deficits in NOD-EAE by suppressing inflammation, demyelination, and neurodegeneration. Moreover, it promoted the functional recovery presumably by promoting the axonal rewiring. Inflammation in the spinal cord occurs prior to the onset of secondary progression, and early inflammation affects the severity of disease progression later^30^. Thus, it would be more effective for preventing secondary progression to start anti-RGM treatment before the onset of secondary progression by suppressing early inflammation. However, previous clinical trials have shown that inhibiting aberrant immune responses is insufficient to treat SPMS^11^. As shown in present study and previous reports, RGMa is implicated in not only inflammation, but also neurodegeneration and neuroregeneration in MS. Thus, humanized anti-RGMa antibody is expected to be the effective treatment for SPMS.

## Materials and methods

### Animals

Nine-week-old female NOD/ShiJcl and 7-week-old female C57BL/6J mice were purchased from CLEA Japan, Inc. and Charles River Laboratories, respectively. Mice were anesthetized by intraperitoneal administration of a mixture of medetomidine hydrochloride (0.3 mg/kg), midazolam (4 mg/kg), and butorphanol (5 mg/kg). All experiments adhered to the guidelines for the care and use of laboratory animals of Osaka University.

### EAE induction

To induce NOD-EAE, we immunized 10-week-old NOD/ShiJcl female mice by subcutaneously injecting 300 μL of an emulsion containing 150 μg MOG_35–55_ peptide (MEVGWYRSPFSRVVHLYRNGK, Sigma Genosys) and 750 μg *Mycobacterium tuberculosis* extract H37Ra (Difco) in incomplete Freund’s adjuvant (IFA, Difco). Next, 200 ng of pertussis toxin (List Biological Laboratories) was intravenously administered to mice at 0 and 48 h after immunization. Twenty days after immunization, 100 or 200 μg of humanized anti-RGMa antibody or 200 μg of palivizumab antibody was intravenously injected into NOD-EAE mice every 3 days. Palivizumab and humanized RGMa antibodies were provided by Mitsubishi Tanabe Pharma Corporation (Osaka, Japan).

To induce targeted EAE, we immunized 8-week-old C57BL/6J female mice by subcutaneous injection of 300 μL of an emulsion containing 100 μg MOG_35–55_ peptide and 500 μg *Mycobacterium tuberculosis* extract H37Ra in IFA. After 18–21 days, we injected a cytokine mixture containing TNF-α (500 ng, R&D Systems) and IFN-γ (2500 U, Peprotech) into the spinal cord. We performed dorsal laminectomy under anesthesia at Th8, and 1 μL of cytokine mixture was injected into the thoracic spinal cord at a depth of 0.5 mm at the midline. We intravenously injected 200 ng of pertussis toxin (List Biological Laboratories) into the mice 0 and 48 h after the cytokine injection. Next, 200 μg of palivizumab or humanized anti-RGMa antibodies was intravenously injected into targeted EAE mice at 10, 14, 18, 22, 26 days after cytokine injection.

We assessed the EAE clinical scores daily according to the following criteria: 0, no abnormalities noted; 0.5, weak tail reflex; 1, loss of tail reflex; 1.5, loss of tail reflex with hind leg inhibition without gait abnormality; 2, partial hind limb paralysis with gait abnormality; 3, complete hind limb paralysis; 4, front and hind limb paralysis; and 5, moribund state. Clinical scores were assessed by investigators who were blinded to the treatments.

### BDA injection

BDA was injected into targeted EAE mice concurrently with the cytokine injection. We injected 10% BDA solution (10,000 MW, Thermo Fisher Scientific) with a glass capillary attached to a microsyringe into the hind limb area of the motor cortex (coordinates from bregma: 0.5 mm posterior/0.5 mm lateral, 0.5 mm posterior/1.0 mm lateral, 1.0 mm posterior/0.5 mm lateral, 1.0 mm posterior/1.0 mm lateral, at a depth of 0.5 mm, 0.4 μL each).

### Western blot analysis

The indicated doses of recombinant human RGMa (R&D Systems) and recombinant mouse RGMa (R&D Systems) were mixed with sample buffer containing 0.3 M Tris-HCl (pH 6.8), 10% sodium dodecyl sulfate (SDS), 20% glycerol, 0.01% bromophenol blue, and 7% 2-mercaptoethanol. Whole spinal cords from intact mice or NOD-EAE mice after 70 days of immunization were lysed in lysis buffer containing 150 mM NaCl, 1% Triton X-100, 20 mM 4-(2-Hydroxyethyl)-1-piperazineethanesulfonic acid (HEPES) (pH 7.4), 10% glycerol, 5 mM ethyenediaminetetraacetic acid (EDTA), and complete protease inhibitor cocktail (Roche Applied Science). After incubation on ice for 30 min, the lysates were centrifuged at 15,000 rpm for 30 min at 4 °C, and the supernatants were mixed with sample buffer. Protein concentrations were determined with a bicinchoninic acid assay kit (Pierce). Recombinant RGMa samples and 37.5 μg of spinal cord sample were resolved by sodium dodecyl sulfate-polyacrylamide gel electrophoresis (SDS-PAGE) and transferred to polyvinylidene fluoride membranes (Millipore). Membranes were blocked with 5% bovine serum albumin in phosphate-buffered saline (PBS) containing 0.05% Tween-20 for 1 h and incubated with humanized anti-RGMa (10 μg/mL) and rabbit anti-β-actin (1:3000, 4970; Cell Signaling Technology) in blocking solution overnight at 4 °C. After washing, the membranes were incubated with horseradish peroxidase-conjugated secondary antibody against human IgG (1:3000; Santa Cruz) or rabbit IgG (1:3000; Cell Signaling Technology) for 1 h. Detection was performed by using Immobilon ECL Ultra western HRP substrate (Millipore) and ChemiDoc Touch MP (Bio-Rad).

### Histological analysis

Mice were anesthetized by intraperitoneal administration of an anesthetic cocktail as previously described and transcardially perfused with ice-cold PBS followed by 4% paraformaldehyde (PFA). The lumbar spinal cord of NOD-EAE mice, thoracic spinal cord at Th8 of targeted EAE mice, and cervical spinal cord at C4−C7 of targeted EAE mice were dissected and fixed with 4% PFA overnight at 4 °C. Tissues were immersed in 30% sucrose in phosphate buffer overnight at 4 °C. The spinal cords were embedded in optimal cutting temperature compound (Tissue-Tek), and sections were prepared at a thickness of 30 μm using a cryostat. For H&E staining, sections were immersed in hematoxylin solution (Muto Kagaku) for 10 min, followed by immersion in water for 7 min. Sections were treated with eosin solution for 10 min and dehydrated step-wise in 70, 80, 95, and 100% ethanol, followed by Histoclear solution (National Diagnostics). The sections were mounted with Entellan New (Merck). For immunohistochemistry, the sections were treated with PBS containing Triton X-100 two times for 10 min and blocked with 3% normal goat/donkey serum in PBS for 1 h at room temperature. The following primary antibodies were used: goat anti-human IgG (1:500, 0855071; MP Biomedicals), goat anti-MBP (1:1000, C-16; Santa Cruz), mouse anti-GFAP (1:1000, G-A-5; Sigma Aldrich), mouse anti-axonal neurofilament (1:1000, SMI-312; Covance), rat anti-CD11b (1:500, M1/70; BD Biosciences), rat anti-CD45 (1:500, 30-F11, BD Biosciences), rabbit anti-neogenin (1:1000, NPB1-89651; Novus Biologicals) and rabbit anti-RGMa (1:1000, 28045; Immuno-Biological Laboratories). Sections were treated with primary antibody at overnight 4 °C. Alexa Fluor 488 or 568-conjugated goat/donkey anti-mouse, rabbit, and rat IgG secondary antibodies were used (1:500, Molecular Probes).

To quantify inflammatory lesions, H&E-stained sections were imaged using brightfield microscopy (Olympus, BX53). The area of the inflammatory lesion was measured with ImageJ software (National Institute of Health). Inflammatory lesions were defined as the accumulation of more than ten cells. For quantification of axonal degeneration, demyelination, and axonal sprouting, images were acquired by confocal microscopy (Olympus, FV1200). To quantify axonal loss in NOD-EAE mice, the area of SMI-312^−^ in the spinal cord section was measured using ImageJ software and normalized to the total area of the spinal cord section. The percentage of axonal loss was calculated by normalizing the SMI-312^−^ area to the total area in control mice. To quantify the remaining axons in targeted EAE mice, the number of SMI-312^+^ axons per mm^2^ in inflammatory lesions of targeted EAE mice was quantified with ImageJ software. To quantify demyelination in NOD-EAE mice, MBP^−^ areas in white matter and total areas of white matter were measured with ImageJ software. The percentage of demyelination was calculated by normalizing value of MBP^−^ area to the total area in control mice. To quantify the demyelinating area in targeted EAE mice, MBP^−^ areas in the inflammatory lesion were measured.

To analyze axonal sprouting of CST, C4−C7 cervical spinal cord sections (50 μm) were treated with 0.1% Triton X-100 two times for 10 min and blocked with 3% normal goat serum in PBS for 1 h at room temperature. Sections were incubated with Alexa 488-conjugated streptavidin (1:500, Thermo Fisher Scientific) in blocking solution for 2 h at room temperature and mounted with fluorescence mounting medium (DAKO). Thirty sections from four segments of C4−C7 spinal cord, spaced 100 μm apart were analyzed. Axons crossing the gray matter were defined as CST collaterals and the number of CST collaterals was counted under a fluorescence microscope. Images were acquired by confocal microscopy and the number of main CST axons was counted with ImageJ software. The total number of CST collaterals was normalized to the number of main CST axons.

### Flow cytometry analysis

Seventy days after immunization, NOD-EAE mice were anesthetized by intraperitoneal administration of an anesthetic cocktail and transcardially perfused with ice-cold PBS to remove blood. Spinal cords were dissected and minced with a scalpel. Tissues were digested with collagenase D (1 mg/mL, Roche Diagnostics) containing 2.5 mM CaCl_2_ at 37 °C for 30 min. Digested tissues were re-suspended in 30% Percoll (GE healthcare). Seventy percent Percoll was overlaid with 30% Percoll, followed by centrifugation at 770 × *g* for 20 min at room temperature. Immune cells were isolated from the interface of the 30/70% Percoll gradients. The cells were incubated with antibody solution diluted in 2% fetal bovine serum in PBS on ice for 30 min. The following fluorescently labeled antibodies were used: FITC-CD8, PE-CD11b, PerCP/Cy5.5-CD45, APC-Ly6G, and Brilliant violet 421-CD4 (all from BioLegend). Data were collected with a FACSVerse fluidics system (BD Biosciences) and analyzed with FlowJo software (Tree Star).

### Statistics

Student’s *t* tests, one-way analyses of variance (ANOVAs) followed by the Tukey–Kramer test, two-way ANOVA followed by the Bonferroni test, Kruskal−Wallis ANOVA test followed by Dunn’s test and the Mann−Whitney test were performed with GraphPad Prism 7 (GraphPad Software). *p* < 0.05 was considered significant. Error bars represent the standard error of the mean.

## References

[1] Trapp BD (1998). Axonal transection in the lesions of multiple sclerosis. N. Engl. J. Med..

[2] Trapp BD, Ransohoff RM, Fisher E, Rudick R (1998). Neurodegeneration in multiple sclerosis: relationship to neurological disability. Neuroscientist.

[3] Weinshenker BG (1989). The natural history of multiple sclerosis: a geographically based study. I. Clinical course and disability. Brain.

[4] Baecher-Allan C, Kaskow BJ, Weiner HL (2018). Multiple sclerosis: mechanism and immunotherapy. Neuron.

[5] Montalban X (2017). Ocrelizumab versus placebo in primary progressive multiple sclerosis. N. Engl. J. Med..

[6] Kappos L (2016). Baseline subgroup characteristics of EXPAND: a phase 3 study of siponimod (BAF312) for the treatment of secondary progressive multiple sclerosis (P3.084). Neurol.

[7] Martinelli Boneschi F, Vacchi L, Rovaris M, Capra R, Comi G (2013). Mitoxantrone for multiple sclerosis. Cochrane Databese Syst. Rev..

[8] Lopez-Diego RS, Weiner HL (2008). Novel therapeutic strategies for multiple sclerosis-a multifaceted adversary. Nat. Rev. Drug Discov..

[9] Filippi M (1996). A spinal cord MRI study of benign and secondary progressive multiple sclerosis. J. Neurol..

[10] Prineas JW (2001). Immunopathology of secondary-progressive multiple sclerosis. Ann. Neurol..

[11] Rovaris M (2006). Secondary progressive multiple sclerosis: current knowledge and future challenges. Lancet Neurol..

[12] Bermel RA, Bakshi R (2006). The measurement and clinical relevance of brain atrophy in multiple sclerosis. Lancet Neurol..

[13] Siebold C, Yamashita T, Monnier PP, Mueller BK, Pasterkamp RJ (2017). RGMs: structural insights, molecular regulation, and downstream signaling. Trends Cell Biol..

[14] Demicheva E (2015). Targeting repulsive guidance molecule A to promote regeneration and neuroprotection in multiple sclerosis. Cell Rep..

[15] Muramatsu R (2011). RGMa modulates T cell responses and is involved in autoimmune encephalomyelitis. Nat. Med..

[16] Tanabe S, Yamashita T (2014). Repulsive guidance molecule-a is involved in Th17-cell-induced neurodegeneration in autoimmune encephalomyelitis. Cell Rep..

[17] Harada K (2018). Inhibition of RGM alleviates symptoms in a rat model of neuromyelitis optica. Sci. Rep..

[18] Basso AS (2008). Reversal of axonal loss and disability in a mouse model of progressive multiple sclerosis. J. Clin. Invest..

[19] Kitayama M, Ueno M, Itakura T, Yamashita T (2011). Activated microglia inhibit axonal growth through RGMa. PLoS ONE.

[20] Hata K (2006). RGMa inhibition promotes axonal growth and recovery after spinal cord injury. J. Cell. Biol..

[21] Kerschensteiner M (2004). Remodeling of axonal connections contributes to recovery in an animal model of multiple sclerosis. J. Exp. Med..

[22] Muramatsu R (2012). Angiogenesis induced by CNS inflammation promotes neuronal remodeling through vessel-derived prostacyclin. Nat. Med..

[23] Raveney BJ (2015). Eomesodeermin-expressing T-helper cells are essential for chronic neuroinflammation. Nat. Commun..

[24] Preziosa P (2011). Intrinsic damage to the major white matter tracts in patients with different clinical phenotypes of multiple sclerosis: a voxelwise diffusion-tensor MR study. Radiology.

[25] Schattling B (2012). TRPM4 cation channel mediates axonal and neuronal degeneration in experimental autoimmune encephalomyelitis and multiple sclerosis. Nat. Med..

[26] Kaneko S (2009). Protecting axonal degeneration by increasing nicotinamide adenine dinucleotide levels in experimental autoimmune encephalomyelitis. J. Neurosci..

[27] Bareyre FM (2004). The injured spinal cord spontaneously forms a new intraspinal circuit in adult rats. Nat. Neurosci..

[28] Rosenzweig ES (2010). Extensive spontaneous plasticity of corticospinal projections after primate spinal cord injury. Nat. Neurosci..

[29] Ceccarelli A (2010). Structural and functional magnetic resonance imaging correlates of motor network dysfunction in primary progressive multiple sclerosis. Eur. J. Neurosci..

[30] Rush CA, MacLean HJ, Freedman MS (2015). Aggressive multiple sclerosis: proposed definition and treatment algorithm. Nat. Rev. Neurol..

